# Prevalence and antimicrobial susceptibility of *Salmonella* in poultry farms and in‐contact humans in Adama and Modjo towns, Ethiopia

**DOI:** 10.1002/mbo3.1067

**Published:** 2020-06-08

**Authors:** Betelhem Dagnew, Haile Alemayehu, Girmay Medhin, Tadesse Eguale

**Affiliations:** ^1^ College of Veterinary Medicine Samara University Samara Ethiopia; ^2^ Aklilu Lemma Institute of Pathobiology Addis Ababa University Addis Ababa Ethiopia

**Keywords:** Adama, antimicrobial susceptibility, human, Modjo, poultry, *Salmonella*

## Abstract

Consumption of contaminated poultry and poultry products represents a common source of nontyphoidal *Salmonella* infection. Little is known on the status of *Salmonella* and their antimicrobial susceptibility in poultry farms in Ethiopia. This study investigated the prevalence, serotype distribution, and antimicrobial susceptibility of nontyphoidal *Salmonella* among poultry farms in Adama and Modjo towns. Three hundred thirty‐four cloacal swabs, 384 fecal droppings of birds, 59 feed, 59 floor swabs, and 36 stools from in‐contact humans were collected and processed for *Salmonella* isolation. Isolates were tested for their susceptibility to 15 antimicrobials using Kirby–Bauer disk diffusion assay. Seventeen (28.8%) of the farms and 24 (2.9%) of the samples from poultry farms and 2.8% (1/36) of stool samples of humans in‐contact with poultry were positive for *Salmonella*. Most of the isolates (*n* = 21) were recovered from fecal droppings of birds while the remaining isolates were recovered from floor swab samples (*n* = 2) and cloacal swab sample (*n* = 1). Only three *Salmonella* serovars: S. Haifa (*n* = 14, 56%), S. Anatum (*n* = 7; 28%), and S. Give (*n* = 4; 16%) were detected. Poultry farms in Adama town, large flock sized farms, and farms that used antimicrobials were significantly associated with the occurrence of *Salmonella* (*p* < .05). Twenty (80%) and 19 (76%) of *Salmonella* isolates were resistant to streptomycin and tetracycline, respectively. Nineteen (76%) of the isolates were resistant to two or more antimicrobials. Detection of multidrug‐resistant strains of *Salmonella* in poultry farms suggests the need for detailed epidemiological and molecular studies to establish sources of acquisition of resistant *Salmonella* strains.

## Graphical abstract

This study investigated the prevalence, serotype distribution, and antimicrobial susceptibility of nontyphoidal *Salmonella* among poultry farms in Ethiopia. Seventeen farms in Adama and Modjo towns (28.8%), 24 samples from poultry farms (2.9%), and 1/36 of stool samples from humans in contact with poultry (2.8%) were positive for *Salmonella*. Twenty (80%) and 19 (76%) of *Salmonella* isolates were resistant to streptomycin and tetracycline, respectively. Nineteen (76%) of the isolates were resistant to two or more antimicrobials.

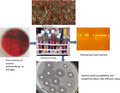

## 1 INTRODUCTION

Foodborne diseases are among the most widespread global public health problems of recent times, and their implication for health and economy is increasingly recognized (Hendriksen et al., 2011). *Salmonella* is one of the most prevalent zoonotic pathogens in both developed and developing countries (Velusamy, Arshak, Korostynska, Oliwa, & Adley, 2010). Nontyphoidal *Salmonella* is mainly acquired through the consumption of contaminated animal‐derived food products and contact with animals. *Salmonella* serovars that are most prevalent in humans are also common in poultry suggesting a possible epidemiologic connection between poultry as a reservoir of *Salmonella* and human infection. For instance, in the United States the most frequently isolated serovars were *S*. Enteritidis and *S*. Heidelberg which were also among the top serovars causing human infection (Foley et al., 2011). *Salmonella* infantis, *S*. Stanley, and *S*. Kentucky originating from poultry meat are also reported to be associated with human salmonellosis in European countries (Antunes, Mourao, Campos, & Peixe, 2016). The previous study from Ethiopia also showed genetic relatedness of *S*. Kentucky isolated from poultry and diarrheic human patients using pulsed‐field gel electrophoresis and isolates displayed similar antimicrobial resistance phenotype suggesting the possibility of poultry to be a source of human infection (Eguale et al., 2018).

The emergence and spread of antimicrobial‐resistant *Salmonella* strains have become a serious health hazard worldwide (Prestinaci, Pezzotti, & Pantosti, 2015). In recent years, *Salmonella* has been reported from several food‐related items in different parts of Ethiopia (Ejo, Garedew, Alebachew, & Worku, 2016; Guchi & Ashenafi, 2010; Ketema et al., 2018). An increase in the resistance of *Salmonella* to commonly used antimicrobials has also been noted in both public health and veterinary sectors in Ethiopia (Eguale, 2018; Eguale et al., 2015; Ketema et al., 2018). The widespread use of antimicrobials in food‐producing animals during rearing has been believed to contribute to the occurrence of *Salmonella* with decreased susceptibility to antimicrobials (Angulo, Johnson, Tauxe, & Cohen, 2000). Multidrug resistance (MDR) to over three antimicrobials has been reported among *Salmonella* isolates from humans and food animals in Ethiopia and elsewhere including resistance to fluoroquinolones and third‐generation cephalosporins, drugs of choice for invasive salmonellosis (Beyene et al., 2011; Eguale et al., 2017; Hendriksen et al., 2011; Kariuki, Gordon, Feasey, & Parry, 2015).

Poultry plays an important role in the livelihood of poor rural households and peri‐urban and urban areas in many developing countries including Ethiopia. During the last few years, small‐scale semi‐intensive poultry farming is flourishing in urban and peri‐urban areas of Ethiopia. This is particularly common in the Modjo and Adama towns of the Oromia region. Most of these small‐scale poultry farms are located near human residential areas suggesting the possibility of transmission of potential pathogens to humans. *Salmonella* is one of the common bacterial pathogens transmitted from poultry and poultry products to humans (FAO; Foley et al., 2011). Integrated surveillance of the common serovars of *Salmonella* circulating in poultry farms and in‐contact humans, as well as their antimicrobial susceptibility status, is useful to envisage possible intervention strategies to control and prevent its widespread impact on public health. A study conducted in central Ethiopia reported that 14.6% of poultry farms in central Ethiopia and 4.7% of the pooled fecal droppings of birds were positive for *Salmonella*. Another study in South Ethiopia that involved three farms reported the detection of *Salmonella* from samples in all the three farms at a rate of 16.7% (Abdi et al., 2017). Small‐scale poultry production is becoming common practice in Adama and Modjo towns from where poultry products are supplied to the population of the two towns and the capital city Addis Ababa (FAO, 2019). Some of these smallholder poultry producers keep chickens in the same compound where they live risking the family members to the transmission of zoonotic pathogens. Despite a large number of poultry farms in Adama and Modjo towns, there are no published data on the presence of *Salmonella* in poultry farms, serovars involved, and their antimicrobial susceptibility. Therefore, this study aimed to determine the prevalence of *Salmonella*, serotype distribution, and to investigate antimicrobial susceptibility of *Salmonella* isolates from poultry and in‐contact humans in Adama and Modjo towns.

## 2 MATERIALS AND METHODS

### 2.1 Study area and study design

This cross‐sectional study was conducted in Adama and Modjo towns, Oromia Regional State, Ethiopia, from November 2017 to May 2018. Adama town is located at about 99 km South East of Addis Ababa. It is located at 8.54°N 39.27°E at an altitude of 1,712 m above sea level. It receives an average rainfall of approximately 600–1,150 mm with annual average minimum and maximum temperatures of 18 and 32°C, respectively. Modjo town is located at 73 km South East of Addis Ababa at an altitude of 1,777 m above sea level. It is located at 8.36°N and 39.7°E. The monthly mean minimum and maximum temperature for Modjo town ranges from 8.5–13.5°C, and 25.6–30.8°C, respectively. Small‐scale urban and peri‐urban poultry production is commonly practiced in these two towns (CSA, 2012). A list of poultry farms in the towns was obtained from District Agricultural Offices, of which representative farms from different localities in the towns were selected for inclusion in the study. All of the farms involved in the current study kept their birds inside and applied the floor system. A total of 59 poultry farms: small farms (*n* = 19: containing less than or equal to 500 birds) and large farms (*n* = 40) having more than 500 birds were involved in the study.

### 2.2 Sample and data collection

A total of 836 poultry‐related samples (334 cloacal swabs, 384 fecal droppings, 59 feed samples, and 59 floor swabs), and 36 stool samples from volunteer farm attendants were collected and investigated. Cloacal swab samples were collected with sterile cotton swabs moistened in 10 ml of sterile buffered peptone water (BPW) by gentle rotation in the cloaca of birds and suspended in 9 ml of BPW. A minimum of 5% of the birds on each farm was sampled. Similarly, floor swabs were collected by swabbing BPW moistened cotton swab on the area of 12 cm by 12 cm surface of the floor where birds were kept. Two swab samples from two opposite corners of the floor were pooled into 10 ml BPW. Fresh fecal droppings (3 pooled droppings) of minimal 5 g were collected using clean disposable gloves into sterile zippered plastic bags. Pooled fecal droppings of a minimum of 5% of the bird population were collected from each farm. Additionally, 5 g of feed sample was also collected from each selected farm into zippered plastic bags. Approximately 1 g of stool sample of human volunteers was also collected into a sterile stool cup with an applicator. All samples were transported to the Microbiology Laboratory of Aklilu Lemma Institute of Pathobiology, Addis Ababa University in an icebox containing ice pack. Also, information such as farm size, type of birds kept in the farm, age of birds, source of birds, and history of antimicrobial use was collected during sample collection using a structured questionnaire.

### 2.3 Isolation, identification, and serotyping of *Salmonella*

For isolation and identification of *Salmonella,* five gm of fecal droppings from birds and 1 g of stool sample from in‐contact humans were pre‐enriched in 45 and 9 ml BPW, respectively, (Oxoid) and incubated for 24 hr at 37°C. Swab samples placed into BPW were also incubated at 37°C for 24 hr. Similarly, 5 g of feed sample was pre‐enriched in 45 ml of BPW and incubated at a similar temperature overnight.

One hundred µl of the pre‐enriched culture was transferred to 10 ml of Rappaport‐Vassiliadis Enrichment Broth (RVB), (Oxoid) and incubated for 24 hr at 42°C. One ml of the suspension was also transferred to 9 ml of Tetrathionate broth (Oxoid) and incubated for 24 hr at 37°C. A loopful of the suspension from both RVB and TTB was then streaked to Xylose Lysine Deoxycholate (XLD) agar plate (Oxoid) selective media and the plates were incubated at 37°C for 24–48 hr. Two presumptive *Salmonella* colonies were picked per plate for further identification and biochemical analysis as described previously (Eguale, 2018). Hence, up to 4 isolates (2 enrichment broths and 2 colonies/XLD) were picked and confirmed by biochemical tests. When *Salmonella* was recovered from the two enrichment broths of a single sample and for two separate colonies picked from a single XLD plate, they were first considered as different strains until the isolates were tested for antimicrobial susceptibility. When isolates from the same sample demonstrated similar antimicrobial susceptibility profile, only one isolate was considered for further analysis.

Isolates showing specific biochemical characteristics of *Salmonella* were further confirmed using *Salmonella* genus‐specific PCR as previously described (Cohen et al., 1993). Serotyping of *Salmonella* isolates was carried out at the National Microbiology Laboratory, Office International des Epizooties (OIE) *Salmonella* Reference Laboratory, Public Health Agency of Canada as described previously based on types of somatic (O) and flagellar (H) antigens of the *Salmonella* isolates using slide agglutination test and microplate agglutination technique, respectively (Ewing, 1986; Popoffn & Minor, 2008; Shipp & Rowe, 1980).

### 2.4 Antimicrobial susceptibility test of isolates

The antimicrobial susceptibility tests of the isolates were performed according to the Clinical Laboratory Standards Institute (CLSI) guideline (CLSI, 2016) using Kirby–Bauer disk diffusion method on Muller–Hinton agar plates (Oxoid, CM0337 Basingstoke). Antimicrobial disks used were amikacin (30 μg), ampicillin (10 μg), cephalothin (30 μg), ceftriaxone (30 μg), chloramphenicol (30 μg), ciprofloxacin (5 μg), gentamycin (10 μg), kanamycin (30 μg), nalidixic acid (30 μg), neomycin (30 μg), nitrofurantoin (100 μg), streptomycin (10 μg), sulfisoxazole (1,000 μg), sulfamethoxazole + trimethoprim (23.75/1.25 μg), tetracycline (30 μg), and trimethoprim (5 μg). Antimicrobial disks used in this study were all from Sensi‐Discs, Becton, Dickinson, and Company. The quality control organism used to conduct antimicrobial susceptibility test was *Escherichia coli* ATCC 25922. The interpretation of the susceptibility test result was based on the CLSI guideline (CLSI, 2016).

### 2.5 Data analysis

*Salmonella* prevalence on poultry farms was calculated as the percentage of farms with one or more *Salmonella* culture positive from any of the poultry‐associated samples among the total poultry farms sampled. A chi‐square test was used to assess the association of various factors with the occurrence of *Salmonella*. Associations were reported as being statistically significant whenever the *p*‐value was <.05.

## 3 RESULTS

### 3.1 Prevalence of *Salmonella* in poultry farms and in‐contact humans

Out of the 59 farms included in the current study, one or more samples were positive in 17 farms, resulting in 28.8% farm‐level occurrence of *Salmonella*. *Salmonella* prevalence on poultry farms was significantly higher in Adama town (50%; 13/26) than in Modjo town (12.1%; 4/33) (*χ* ^2^ = 10.2, *p* < .001). All the 24 *Salmonella* positive samples from poultry farms were obtained from farms using antimicrobials for prophylaxis or therapeutic purposes. None of the farms with no recent history of use of antimicrobials were positive for *Salmonella*. There was a significant difference in *Salmonella* occurrence among farms with different flock size (*p* = .033). Isolation was more common in large flock sized farms. There was no significant difference in *Salmonella* prevalence between farms which kept layers and those which kept broilers (*p* > .05). The occurrence of *Salmonella* was not significantly associated with the origin and age of birds contained in farms (*p* > .05) (Table 1).

**TABLE 1:** Farm‐level prevalence and associated risk factors of *Salmonella* in Adama and Modjo towns

Characteristics	Categories	No. of farms examined	No. (%) of positive farms	Chi‐square	*p*‐value
Towns	Adama	26	13 (50)	10.2	.001
	Modjo	33	4 (12.1)		
Flock size	Small^a^	19	2 (10.5)	4.6	.033
	Large^b^	40	15 (37.5)		
Antimicrobial use	Yes	46	17 (37)	6.8	.009
	No	13	0 (0.00)		
Type of bird	Layer	43	11 (25.6)	0	.369
	Broiler	16	6 (37.5)		
Source of birds	Farm A	17	5 (29.4)	2.1	.354
	Farm B	31	7 (22.6)		
	Unknown	11	5 (45.5)		
Age of chickens	<6 months	25	9 (36)	1.2	.546
	6–14 months	23	5 (21.7)		
	>14 months	11	3 (27.3)		
Total		59	17 (28.8)		

^a^ Small ≤ 500 birds.

^b^ Large > 500.

Out of all 872 samples cultured for *Salmonella* (836 from various samples in poultry farms and 36 from human stool samples), 25 samples were confirmed *Salmonella* positive. Only 24 samples (2.9%) of 836 samples collected on poultry farms were *Salmonella* positive. Out of the 384 pooled fecal droppings investigated in the current study, 21(5.5%) were *Salmonella* positive: 17 from 12 farms in Adama and 4 from 4 farms in Modjo town. On the other hand, out of 334 cloacal swabs collected, only one sample (0.3%) from the farm in Adama town was positive for *Salmonella*. Of the 59 pooled floor swabs tested, only 2 (3.4%) were positive for *Salmonella* both of which were from farms in Adama town. Fecal droppings of birds in these farms were also positive (Table 2). None of the feed samples obtained from poultry farms were positive for *Salmonella*. A stool sample was collected from 36 volunteer individuals working in the poultry farms, and only 1 person (2.8%) from a farm in Adama town was positive for *Salmonella*.

**TABLE 2:** Sample level prevalence of *Salmonella* in poultry farms and associated risk factors in Adama and Modjo towns

Characteristics	Categories	No. of samples examined	No. (%) of positive samples	Chi‐square	*p*‐value
Towns	Adama	354	20 (5.7)	17.0	<.001
	Modjo	482	4 (0.8)		
Age in months	<6	388	14 (3.6)	1.7	.427
	6–14	272	7 (2.6)		
	≥14	176	3 (1.7)		
Source of birds	Farm A	297	5 (1.7)	4.8	.089
	Farm B	365	10 (2.7)		
	Unknown	176	9 (5.2)		
Type of commodity	Layer	614	17 (2.8)	0.1	.769
	Broiler	222	7 (3.2)		
Type of sample	Cloacal swab	334	1 (0.3)	19.0	<.001
	Feces	384	21 (5.5)		
	Feed	59	0 (0.0)		
	Floor swab	59	2 (3.4)		
Total		836	24 (2.9)		

### 3.2 *Salmonella* serotype distribution

Only three serovars (i.e., *S*. Haifa, *S*. Anatum, and *S*. Give) all belonging to *Salmonella enterica* subspecies *enterica* were detected. In the present study, only one serovar was detected per farm. Nine of the 17 positive farms (52.9%), 5(29.4%), and 3(17.7%) farms were positive for *S*. Haifa, *S*. Anatum, and *S*. Give, respectively. Most of *S*. Haifa serovars (*n* = 13; 92.9%), all of the four *S*. Give (100%) and 4 (57.1%) of *S*. Anatum were isolated from farms in Adama town including a single human isolate. The single isolate obtained from the in‐contact human stool sample was also *S*. Anatum which was the same as the serovar isolated from feces of birds in the same farm. There was no linkage of specific serovar with the source of birds as the three serovars were distributed across different farms irrespective of the origin of birds. Of the seven farms, that received day‐old chickens from a particular chicken multiplication farm, four farms were positive for *S*. Haifa, two for *S*. Anatum, and one for *S*. Give. Similarly, from five farms that received chickens from another multiplication farm, two of them were positive for *S*. Anatum the other two for *S*. Haifa, and one farm for *S*. Give.

### 3.3 Antimicrobial susceptibility of *Salmonella* isolates

None of the isolates obtained from a single sample in this study demonstrated a difference in antimicrobial susceptibility profile, and all *Salmonella* isolates from a single sample were considered only once in all analyses. One or more isolates demonstrated resistance to streptomycin, tetracycline, nitrofurantoin, sulfisoxazole, neomycin, and kanamycin. The percentage of isolates resistant to streptomycin, tetracycline, nitrofurantoin, sulfisoxazole, neomycin, and kanamycin accounted for 20 (80%), 19 (76%), 11 (44%), 8 (32%), 6 (24%), and 3 (12%), respectively (Table 3). Thirteen out of 14 (92.9%) and 14 (100%) of *S*. Haifa isolates were resistant to streptomycin and tetracycline, respectively.

**TABLE 3:** Rate of occurrence of resistance to antimicrobials among *Salmonella* serovars isolated from poultry farms

Antimicrobials tested	*S*. Anatum (*n* = 7)	*S*. Give (*n* = 4)	*S*. Haifa (*n* = 14)	Total No. (%) resistant
	No. (%) resistant	No. (%) resistant	No. (%) resistant	
Amikacin	0 (0)	0 (0)	0 (0)	0 (0)
Ampicillin	0 (0)	0 (0)	0 (0)	0 (0)
Cephalothin	0 (0)	0 (0)	0 (0)	0 (0)
Ceftriaxone	0 (0)	0 (0)	0 (0)	0 (0)
Chloramphenicol	0 (0)	0 (0)	0 (0)	0 (0)
Ciprofloxacin	0 (0)	0 (0)	0 (0)	0 (0)
Gentamicin	0 (0)	0 (0)	0 (0)	0 (0)
Kanamycin	0 (0)	0 (0)	3 (21.4)	3 (12)
Nalidixic acid	0 (0)	0 (0)	0 (0)	0 (0)
Neomycin	1 (14.3)	0 (0)	5 (35.7)	6 (24)
Nitrofurantoin	2 (28.6)	4 (100)	5 (35.7)	11 (44)
Streptomycin	7 (100)	0 (0)	13 (92.9)	20 (80)
Sulfisoxazole	6 (85.7)	0 (0)	2 (14.3)	8 (32)
Sulfamethoxazole + trimethoprim	0 (0)	0 (0)	0 (0)	0 (0)
Tetracycline	5 (71.4)	0 (0)	14 (100)	19 (76)
Trimethoprim	0 (0)	0 (0)	0 (0)	0 (0)

Nineteen (76%) of the isolates were resistant to two or more of the antimicrobials tested, while resistance to three or more antimicrobials was detected in 15 (60%) of the total isolates. Resistance to four or more antimicrobials was detected in 10 (40%) of the isolates. The single isolate obtained from humans was resistant to 4 of the antimicrobials tested. The resistance pattern of the isolate obtained from humans was almost similar to the two *Salmonella* isolates obtained from the feces of chickens in the farm in which the person was working. They shared a common resistance phenotype to streptomycin, sulfisoxazole, and tetracycline. From seven isolates belonging to serovar *S*. Anatum isolated from five poultry farms (2 from Adama and 3 from Modjo), five of them exhibited resistance to streptomycin, sulfisoxazole, and tetracycline (Table 4).

**TABLE 4:** Resistance pattern of *Salmonella* isolated from poultry farms and in‐contact human in Adama and Modjo towns

No.	Study site	Farm code	Isolate code	Type of sample	Serotype	Resistance pattern
1	Adama	APF1	AP 10	Feces	Give	Fm
2	Adama	APF1	AP 12	Feces	Give	Fm
3	Adama	APF4	AP 58	Feces	Haifa	K, S, Te
4	Adama	APF7	AP 89	Feces	Haifa	K, S, Te
5	Adama	APF7	AP 96	Feces	Haifa	N, Fm, S, Te
6	Adama	APF9	AP 112	Feces	Haifa	K, S, Te
7	Adama	APF10	AP 122	Feces	Anatum	S, Su, Te
8	Adama	APF10	AP 124	Feces	Anatum	S, Su, Te
9	Adama	APF11	AP 140	Feces	Haifa	N, Fm, S, Su, Te
10	Adama	APF11	AP 141	Feces	Haifa	S, Te
11	Adama	APF12	AP 153	Feces	Haifa	S, Su, Te
12	Adama	APF13	AP 161	Feces	Haifa	S, Te
13	Adama	APF13	AP 162	Feces	Haifa	N, Fm, S, Te
14	Adama	APF14	AP 170	Feces	Haifa	N, Fm, S, Te
15	Adama	APF17	AP 196	CS	Anatum	Fm, S, Su
16	Adama	APF19	AP 218	Feces	Haifa	N, Fm, S, Te
17	Adama	APF22	AP 233	Feces	Give	Fm
18	Adama	APF23	AP 262	Feces	Give	Fm
19	Adama	APF10	AH 04	Stool	Anatum	Fm, S, Su, Te
20	Adama	APF7	Fl 7	Fl	Haifa	S, Te
21	Adama	APF14	Fl 14	Fl	Haifa	S, Te
22	Modjo	MPF19	MP 192	Feces	Anatum	S
23	Modjo	MPF31	MP 374	Feces	Anatum	S, Su, Te
24	Modjo	MPF33	MP410	Feces	Anatum	N, S, Su, Te
25	Modjo	MPF18	MP 191	Feces	Haifa	Te

Note

Amp = ampicillin, Cf = cephalothin, Cro = ceftriaxone, Cip = ciprofloxacin, Gm = gentamicin, K = kanamycin, Tmp = trimethoprim, S = Streptomycin, Sxt = sulfamethoxazole+trimethoprim, Te = tetracycline, Su = sulfisoxazole, Fm = nitrofurantoin, Na = nalidixic acid, *N* = neomycin; CS = cloacal swab, Fl = floor swab.

A review of data on the type of antimicrobials that farms used within 6 months before the survey showed that over half of the poultry farms (*n* = 30, 50.8%) used one or more antimicrobials whereas 29 (49.2%) of the farms reported that they did not use any antimicrobial. Oxytetracycline was the most common antimicrobial used in the poultry farms where 23 of the 30 farms (76.7%) reported its use. In 11 of these farms, it was used alone whereas; in 12 farms, it was reported to be used together with sulfonamides. Out of these farms, 14 (60.9%) were positive for *Salmonella*. Four of these farms were using oxytetracycline alone and 10 of them were using oxytetracycline + sulfonamide. The second commonly used antimicrobial group was those agents belonging to the sulfonamide group used in 16 (53.3%) of the 30 farms using antimicrobials of which 11 (68.8%) of the farms were positive for *Salmonella*. One of these farms was using sulfonamide alone and 10 of them were using sulfonamide together with oxytetracycline (Table 5). In addition to the antimicrobial preparations intended for poultry use, two farms (3.4%) also reported the use of ciprofloxacin tablets intended for human use.

**TABLE 5:** Recent use of antimicrobials and the occurrence of *Salmonella* in poultry farms

Type of antimicrobials used	No. of farms	No.(%) of *Salmonella* positive farms
Oxytetracycline	11	4 (36.4)
Sulfonamide	6	2 (33.3)
Oxytetracycline + Sulfonamide	12	10 (83.3)
Enrofloxacin	1	1 (100)
Ciprofloxacin (Human preparation)	2	1 (50)
Did not use antimicrobials	29	0 (0.0)

## 4 DISCUSSION

*Salmonella* prevalence on poultry farms in the two towns Modjo and Adama is much lower than the prevalence reported in Nigeria (Fagbamila et al., 2017) and Morocco (Ziyate et al., 2016) but higher than the prevalence reported from Canada (Lebert et al., 2018) and central Ethiopia (Eguale, 2018). The possible reason for such variation could be due to differences in the management system, such as poultry housing system, farm size, and hygienic practices. All of the farms involved in the current study kept birds in floor‐based housing. Keeping birds in cages leads to the persistence of *Salmonella* in poultry farms compared to keeping on the floor due to poor cleaning standards and disinfection on farms that use cages (Davies & Breslin, 2003). The occurrence of *Salmonella* from various samples in poultry farms in the current study is lower than a study from southern Ethiopia which reported high occurrence of *Salmonella* in various samples from 3 poultry farms (Abdi et al., 2017) and a recent study reported very low prevalence of *Salmonella* from fecal droppings of birds in central Ethiopia which is closely in line with the finding of the current study (Eguale, 2018).

The probable reason for the high level of occurrence of *Salmonella* in poultry farms in Adama town compared to Modjo town may be due to high farm to farm transmission in farms in Adama town as most of the poultry farms in the town were small scale found in the same compound with the human residential area or close to each other. The high *Salmonella* contamination rate of fecal droppings compared to other samples agrees with the previous study conducted in Morocco in which the highest detection rate of *Salmonella* was from fecal droppings followed by floor swab/dust samples and cloacal swabs (Ziyate et al., 2016). The high rate of recovery of *Salmonella* in fecal droppings could be because fecal droppings contain much more intestinal content for analysis than a cloacal swab. The fact that none of the feed samples investigated in the current study were positive for *Salmonella* suggests the safety of feed used in the poultry farms in the study area with regard to *Salmonella* contamination. However, as the number of feed samples investigated in the current study is small, this finding may not represent all poultry feed used in the study area.

In line with previous findings (Eguale, 2018; Mollenhorst, van Woudenbergh, Bokkers, & de Boer, 2005), isolation of *Salmonella* was more common in large flock sized farms compared to small‐sized farms. *Salmonella* was detected only in farms that use antimicrobials whereas none was detected in farms with no usage history of antimicrobials. The possible reason could be due to the selection pressure imposed on other susceptible bacterial species in the farm environment where antimicrobials are used, escalating the chance of multiplication of resistant *Salmonella* strains hence increasing the rate of detection. Most of the *Salmonella* isolates in the current study were resistant to antimicrobials particularly tetracycline, the antimicrobial agent commonly used in the poultry farms in the study area. Similarly, a recent study in central Ethiopia (Eguale, 2018) detected *Salmonella* only from poultry farms that used antimicrobials.

The dominance of *S*. Haifa in the current study is contrary to the very low prevalence of *S*. Haifa in poultry farms reported by the recent study conducted in central Ethiopia where *S*. Saintpaul was the dominant serovar (Eguale, 2018). *Salmonella* Haifa has been reported from various food animals in Ethiopia (Alemu & Zewde, 2012; Ketema et al., 2018; Zewdu & Cornelius, 2009). The second frequently isolated serovar in the current study was *S*. Anatum. Detection of only 3 serovars with closely related antimicrobial susceptibility profile from 17 *Salmonella* positive farms suggests the possibility of contamination across farms and acquiring *Salmonella* strains from breeding farms. Majority of the farms involved in the current study obtained day‐old chickens, pullets, and broilers from a few common chicken breeding centers.

The majority of the farm attendants did not consent to give stool samples for investigation and the occurrence of *Salmonella* among the consented individuals was low. One possible explanation for this low level of positivity could be linked to the small sample size of the study participants. Proper hygienic practices could be another possible explanation as the majority of the farm attendants wash their hands with detergent after getting contact with birds (data not shown) which might have enabled them not to get infected with *Salmonella*. The single *S*. Anatum detected from in‐contact humans in the current study might be due to contamination from the farm environment suggested because of serovar similarity and related antimicrobial resistance pattern with *Salmonella* isolates obtained from the same farm.

The high percentage of *Salmonella* isolates resistant to streptomycin in the current study is in line with the previous reports among isolates from poultry (Eguale, 2018) and other food animals (Ketema et al., 2018). The high rate of resistance to streptomycin could be because it was one of antimicrobials commonly used previously in veterinary medicine in Ethiopia (Beyene, Endalamaw, Tolossa, & Feyisa, 2015) which might have contributed to selection of resistant strains carrying various plasmid‐mediated resistant genetic markers.

The high rate of resistance to tetracycline among *Salmonella* isolates is also in agreement with other studies conducted on *Salmonella* isolates from food animals in Ethiopia (Abdi et al., 2017; Eguale et al., 2014) and elsewhere (Sodagari, Mashak, & Ghadimianazar, 2015). Oxytetracycline has been one of the most overused antimicrobials for treatment and prophylaxis from day‐old chicks to layers in the current study and is one of the commonly used antimicrobials in veterinary medicine in the country (Eguale et al., 2016). The frequent use of this antimicrobial in most of the farms could have contributed to selection of isolates resistant to tetracycline. Susceptibility to certain antimicrobials appears to be related to serovar type. For instance, all *S*. Give isolated from two different farms in Adama town were resistant to nitrofurantoin and all of the 14 *S*. Haifa were resistant to tetracycline irrespective of the difference in the farms involved. The fact that the resistance pattern of isolates was serovar dependent irrespective of the source of isolation could be due to the circulation of related strains across farms or acquisition of resistant trains or resistance genetic markers from breeding farms.

Interestingly, unlike other previous reports from Ethiopia, resistance to beta‐lactam drugs like ampicillin and first‐generation cephalosporin (cephalothin) was not observed in the current study. For instance, a recent study reported 42.3% of resistance to ampicillin and 46.2% resistance to cephalothin in *Salmonella* isolates from poultry in Ethiopia (Eguale, 2018). Another recent study in southern Ethiopia reported 97.8% of resistance to ampicillin (Abdi et al., 2017) in *Salmonella* isolates from poultry farms. The reason why relatively low resistance rates were observed in the present study compared to previous studies could be due to new strains of *Salmonella* serovars circulating in the study farms without genetic markers conferring resistance to beta‐lactam antimicrobials or loss of genetic markers responsible for beta‐lactam resistance because of less use of beta‐lactam antimicrobials in the farms in the current study. None of the farms in the current study reported the use of beta‐lactams whereas in a previous study 29.2% of the poultry farms reported the use of amoxicillin (Eguale, 2018).

Susceptibility to amikacin, chloramphenicol, gentamicin, and ceftriaxone could be due to minimal or no use of these antimicrobials in the study area. The occurrence of resistance to two or more antimicrobials in the majority of the isolates in the current study is in line with the previous findings (Rianatou, Fofana, Seydi, & Akakpo, 2006; Eguale, 2018). The reason for the high rate of MDR might be attributed to the level of use of specific antibacterial agents in the poultry farms which affected the level of selection pressure that contributed to maintaining resistance genes in the bacterial population (Marshall & Levy, 2011).

## 5 CONCLUSION

High *Salmonella* occurrence on poultry farms and a high proportion of MDR *Salmonella* isolates in the current study implies significant public health risk of poultry‐associated salmonellosis. Imprudent use of antimicrobials in the farms can favor the continual occurrence of antimicrobial‐resistant *Salmonella* within the human and animal population. Further detailed epidemiological and molecular studies are essential to identify sources of acquisition of resistant *Salmonella* strains and resistance genetic markers among poultry, poultry products, and humans in the country.

## 6 ETHICS STATEMENT

The study was approved by the Institutional Review Board of Aklilu Lemma Institute of Pathobiology, Addis Ababa University (Minutes Ref No.: ALIPB/IRB/006/2017/18). Individual oral informed consent was obtained from study participants.

## CONFLICT OF INTERESTS

None declared.

## AUTHORS CONTRIBUTION

**Betelhem Dagnew:** Data curation (equal); formal analysis (equal); investigation (lead); methodology (equal). **Haile Alemayehu:** Formal analysis (equal); investigation (equal); methodology (equal); supervision (equal); writing – review & editing (equal). **Girmay Medhin:** Formal analysis (equal); software (lead); supervision (equal); writing – review & editing (equal). **Tadesse Eguale:** Conceptualization (lead); data curation (equal); formal analysis (equal); funding acquisition (lead); investigation (equal); methodology (equal); project administration (lead); resources (lead); supervision (lead); validation (equal); visualization (equal); writing – original draft (equal); writing – review & editing (equal).

## Data Availability

The data used to support the findings of this study are included in the article.

## References

[mbo31067-bib-0001] Abdi, R. D. , Mengstie, F. , Beyi, A. F. , Beyene, T. , Waktole, H. , Mammo, B. , … Abunna, F. (2017). Determination of the sources and antimicrobial resistance patterns of Salmonella isolated from the poultry industry in Southern Ethiopia. BMC Infectious Diseases, 17(1), 352 10.1186/s12879-017-2437-2 28521744 PMC5437651

[mbo31067-bib-0002] Alemu, S. , & Zewde, B. M. (2012). Prevalence and antimicrobial resistance profiles of *Salmonella enterica* serovars isolated from slaughtered cattle in Bahir Dar, Ethiopia. Tropical Animal Health and Production, 44(3), 595–600. 10.1007/s11250-011-9941-y 21814750

[mbo31067-bib-0003] Angulo, F. J. , Johnson, K. R. , Tauxe, R. V. , & Cohen, M. L. (2000). Origins and consequences of antimicrobial‐resistant nontyphoidal Salmonella: Implications for the use of fluoroquinolones in food animals. Microbial Drug Resistance, 6(1), 77–83. 10.1089/mdr.2000.6.77 10868811

[mbo31067-bib-0004] Antunes, P. , Mourao, J. , Campos, J. , & Peixe, L. (2016). Salmonellosis: The role of poultry meat. Clinical Microbiology and Infection: The Official Publication of the European Society of Clinical Microbiology and Infectious Diseases, 22(2), 110–121. 10.1016/j.cmi.2015.12.004 26708671

[mbo31067-bib-0005] Bada‐Alambedji, R. , Fofana, A. , Seydi, M. , & Akakpo, A. J. (2006). Antimicrobial resistance of Salmonella isolated from poultry carcasses in Dakar (Senegal). Brazilian Journal of Microbiology, 37(4), 510–515. 10.1590/S1517-83822006000400020

[mbo31067-bib-0006] Beyene, G. , Nair, S. , Asrat, D. , Mengistu, Y. , Engers, H. , & Wain, J. (2011). Multidrug resistant Salmonella concord is a major cause of salmonellosis in children in Ethiopia. Journal of Infection in Developing Countries, 5, 23–33. 21330737 10.3855/jidc.906

[mbo31067-bib-0007] Beyene, T. , Endalamaw, D. , Tolossa, Y. , & Feyisa, A. (2015). Evaluation of rational use of veterinary drugs especially antimicrobials and anthelmintics in Bishoftu, Central Ethiopia. BMC Research Notes, 8, 482 10.1186/s13104-015-1466-4 26415926 PMC4584433

[mbo31067-bib-0008] CLSI (2016). Performance standards for antimicrobial susceptibility testing; Twenty‐sixth informational supplement. CLSI document M100‐S26. 26th ed. Wayne, PA: Clinical and Laboratory Standards Institute.

[mbo31067-bib-0009] Cohen, N. D. , Neibergs, H. L. , McGruder, E. D. , Whitford, H. W. , Behle, R. W. , Ray, P. M. , & Hargis, B. M. (1993). Genus‐specific detection of salmonellae using the polymerase chain reaction (PCR). Journal of Veterinary Diagnostic Investigation, 5(3), 368–371. 8373849 10.1177/104063879300500311

[mbo31067-bib-0010] CSA (2012). The 2012 population projection from 2007 population census. Addis Ababa, Ethiopia: Central Statistical Agency of the Federal Democratic Republic of Ethiopia.

[mbo31067-bib-0011] Davies, R. , & Breslin, M. (2003). Observations on Salmonella contamination of commercial laying farms before and after cleaning and disinfection. The Veterinary Record, 152(10), 283–287. 12650470 10.1136/vr.152.10.283

[mbo31067-bib-0012] Eguale, T. (2018). Non‐typhoidal Salmonella serovars in poultry farms in central Ethiopia: Prevalence and antimicrobial resistance. BMC Veterinary Research, 14(1), 217 10.1186/s12917-018-1539-4 29980208 PMC6035434

[mbo31067-bib-0013] Eguale, T. , Asrat, D. , Alemayehu, H. , Nana, I. , Gebreyes, W. A. , Gunn, J. S. , & Engidawork, E. (2018). Phenotypic and genotypic characterization of temporally related nontyphoidal Salmonella strains isolated from humans and food animals in central Ethiopia. Zoonoses and Public Health, 65(7), 766–776. 10.1111/zph.12490 29984468

[mbo31067-bib-0014] Eguale, T. , Birungi, J. , Asrat, D. , Njahira, M. N. , Njuguna, J. , Gebreyes, W. A. , … Engidawork, E. (2017). Genetic markers associated with resistance to beta‐lactam and quinolone antimicrobials in non‐typhoidal Salmonella isolates from humans and animals in central Ethiopia. Antimicrobial Resistance and Infection Control, 6, 13 10.1186/s13756-017-0171-6 28105330 PMC5240271

[mbo31067-bib-0015] Eguale, T. , Engidawork, E. , Gebreyes, W. A. , Asrat, D. , Alemayehu, H. , Medhin, G. , … Gunn, J. S. (2016). Fecal prevalence, serotype distribution and antimicrobial resistance of Salmonellae in dairy cattle in central Ethiopia. BMC Microbiology, 16(1), 1–11. 10.1186/s12866-016-0638-2 26879347 PMC4754838

[mbo31067-bib-0016] Eguale, T. , Gebreyes, W. A. , Asrat, D. , Alemayehu, H. , Gunn, J. S. , & Engidawork, E. (2015). Non‐typhoidal Salmonella serotypes, antimicrobial resistance and co‐infection with parasites among patients with diarrhea and other gastrointestinal complaints in Addis Ababa. Ethiopia. BMC Infectious Diseases, 15, 497 10.1186/s12879-015-1235-y 26537951 PMC4634906

[mbo31067-bib-0017] Eguale, T. , Marshall, J. , Molla, B. , Bhatiya, A. , Gebreyes, W. A. , Engidawork, E. , … Gunn, J. S. (2014). Association of multicellular behaviour and drug resistance in *Salmonella enterica* serovars isolated from animals and humans in Ethiopia. Journal of Applied Microbiology, 117(4), 961–971. 10.1111/jam.12579 24934091 PMC4165716

[mbo31067-bib-0018] Ejo, M. , Garedew, L. , Alebachew, Z. , & Worku, W. (2016). Prevalence and antimicrobial resistance of Salmonella isolated from animal‐origin food items in Gondar, Ethiopia. Journal of Biomedicine and Biotechnology. (Vol. 8). 10.1155/2016/4290506 PMC5198084 28074185

[mbo31067-bib-0019] Ewing, W. H. (Ed.) (1986). Serological identification of *Salmoenella* (4th ed., pp. 201–238). New York, NY: Elsevier Science Publishing Co., Inc.

[mbo31067-bib-0020] Fagbamila, I. O. , Barco, L. , Mancin, M. , Kwaga, J. , Ngulukun, S. S. , Zavagnin, P. , … Muhammad, M. (2017). Salmonella serovars and their distribution in Nigerian commercial chicken layer farms. PLoS One, 12(3), e0173097 10.1371/journal.pone.0173097 28278292 PMC5344354

[mbo31067-bib-0021] FAO (2008). Review of the new features of the Ethiopian poultry sector biosecurity implications http://www.fao.org/3/al837e/al837e00.pdf.

[mbo31067-bib-0022] FAO (2019). Poultry Sector Ethiopia. FAO animal production and health livestock country reviews. Retrieved from http://www.fao.org/3/ca3716en/ca3716en.pdf

[mbo31067-bib-0023] Foley, S. L. , Nayak, R. , Hanning, I. B. , Johnson, T. J. , Han, J. , & Ricke, S. C. (2011). Population dynamics of *Salmonella enterica* serotypes in commercial egg and poultry production. Applied and Environmental Microbiology, 77(13), 4273–4279. 10.1128/AEM.00598-11 21571882 PMC3127710

[mbo31067-bib-0024] Guchi, B. , & Ashenafi, M. (2010). Microbial load, prevalence and antibiograms of salmonella and Shigella in lettuce and green peppers. Ethiopian Journal of Health Sciences, 20(1), 41–48. 22434959 10.4314/ejhs.v20i1.69431 PMC3275899

[mbo31067-bib-0025] Hendriksen, R. S. , Vieira, A. R. , Karlsmose, S. , Wong, D. M. L. F. , Jensen, A. B. , Wegener, H. C. , & Aarestrup, F. M. (2011). Global monitoring of Salmonella serovar distribution from the World Health Organization Global Foodborne Infections Network Country Data Bank: Results of quality assured laboratories from 2001 to 2007. Foodborne Pathogens and Disease, 8(8), 887–900. 10.1089/fpd.2010.0787 21492021

[mbo31067-bib-0026] Kariuki, S. , Gordon, M. A. , Feasey, N. , & Parry, C. M. (2015). Antimicrobial resistance and management of invasive Salmonella disease. Vaccine, 33(Suppl. 3), C21–C29. 10.1016/j.vaccine.2015.03.102 25912288 PMC4469558

[mbo31067-bib-0027] Ketema, L. , Ketema, Z. , Kiflu, B. , Alemayehu, H. , Terefe, Y. , Ibrahim, M. , & Eguale, T. (2018). Prevalence and antimicrobial susceptibility profile of Salmonella serovars isolated from slaughtered cattle in Addis Ababa, Ethiopia. BioMed Research International, 2018, 9794869 10.1155/2018/9794869 30533445 PMC6247655

[mbo31067-bib-0028] Lebert, L. , Martz, S. L. , Janecko, N. , Deckert, A. E. , Agunos, A. , Reid, A. , … McEwen, S. A. (2018). Prevalence and antimicrobial resistance among *Escherichia coli* and Salmonella in Ontario smallholder chicken flocks. Zoonoses and Public Health, 65(1), 134–141. 10.1111/zph.12381 28766871

[mbo31067-bib-0029] Marshall, B. M. , & Levy, S. B. (2011). Food animals and antimicrobials: Impacts on human health. Clinical Microbiology Reviews, 24(4), 718–733. 10.1128/CMR.00002-11 21976606 PMC3194830

[mbo31067-bib-0030] Mollenhorst, H. , van Woudenbergh, C. J. , Bokkers, E. G. , & de Boer, I. J. (2005). Risk factors for Salmonella enteritidis infections in laying hens. Poultry Science, 84(8), 1308–1313. 10.1093/ps/84.8.1308 16156216

[mbo31067-bib-0031] Popoffn, M. Y. , & Minor, L. (2008). Antigenic formulae of the Salmonella serovars (8th ed.). Paris, France: WHO Collaborating Center for Reference and Research on Salmonella.

[mbo31067-bib-0032] Prestinaci, F. , Pezzotti, P. , & Pantosti, A. (2015). Antimicrobial resistance: A global multifaceted phenomenon. Pathogens and Global Health, 109(7), 309–318. 10.1179/2047773215Y.0000000030 26343252 PMC4768623

[mbo31067-bib-0033] Shipp, C. R. , & Rowe, B. (1980). A mechanised microtechnique for salmonella serotyping. Journal of Clinical Pathology, 33(6), 595–597. 10.1136/jcp.33.6.595 7400362 PMC1146150

[mbo31067-bib-0034] Sodagari, H. R. , Mashak, Z. , & Ghadimianazar, A. (2015). Prevalence and antimicrobial resistance of Salmonella serotypes isolated from retail chicken meat and giblets in Iran. Journal of Infection in Developing Countries, 9(5), 463–469. 10.3855/jidc.5945 25989165

[mbo31067-bib-0035] Velusamy, V. , Arshak, K. , Korostynska, O. , Oliwa, K. , & Adley, C. (2010). An overview of foodborne pathogen detection: In the perspective of biosensors. Biotechnology Advances, 28(2), 232–254. 10.1016/j.biotechadv.2009.12.004 20006978

[mbo31067-bib-0036] Zewdu, E. , & Cornelius, P. (2009). Antimicrobial resistance pattern of Salmonella serotypes isolated from food items and personnel in Addis Ababa, Ethiopia. Tropical Animal Health and Production, 41(2), 241–249. 10.1007/s11250-008-9181-y 18516698

[mbo31067-bib-0037] Ziyate, N. , Karraouan, B. , Kadiri, A. , Darkaoui, S. , Soulaymani, A. , & Bouchrif, B. (2016). Prevalence and antimicrobial resistance of Salmonella isolates in Moroccan laying hens farms. The Journal of Applied Poultry Research, 25(4), 539–546. 10.3382/japr/pfw036

